# Arrhythmogenic actions of the Ca^2+^ channel agonist FPL-64716 in Langendorff-perfused murine hearts

**DOI:** 10.1113/expphysiol.2008.044669

**Published:** 2008-10-31

**Authors:** Nina S Ghais, Yanmin Zhang, Andrew A Grace, Christopher L-H Huang

**Affiliations:** 1 Physiological Laboratory, University of CambridgeDowning Street, Cambridge CB2 3EG, UK; 2 Department of Biochemistry, University of CambridgeTennis Court Road, Cambridge CB2 1QW, UK

## Abstract

The experiments explored the extent to which alterations in L-type Ca^2+^ channel-mediated Ca^2+^ entry triggers Ca^2+^-mediated arrhythmogenesis in Langendorff-perfused murine hearts through use of the specific L-type Ca^2+^ channel modulator FPL-64716 (FPL). Introduction of FPL (1 μm) resulted in a gradual development (>10 min) of diastolic electrical events and alternans in spontaneously beating hearts from which monophasic action potentials were recorded. In regularly paced hearts, they additionally led to non-sustained and sustained ventricular tachycardia (nsVT and sVT). Programmed electrical stimulation (PES) resulted in nsVT and sVT after 5–10 and >10 min perfusion, respectively. Pretreatments with nifedipine, diltiazem and cyclopiazonic acid abolished arrhythmogenic tendency induced by subsequent introduction of FPL, consistent with its dependence upon both extracellular Ca^2+^ entry and the degree of filling of the sarcoplasmic reticular Ca^2+^ store. Values for action potential duration at 90% repolarization when any of these agents were applied to FPL-treated hearts became indistinguishable from those shown by untreated control hearts, in contrast to earlier reports of their altering in long QT syndrome type 3 and hypokalaemic murine models for re-entrant arrhythmogenesis. These arrhythmic effects instead correlated with alterations in Ca^2+^ homeostasis at the single-cell level found in investigations of the effects of both FPL and the same agents in regularly stimulated fluo−3 loaded myocytes. These findings are compatible with a prolonged extracellular Ca^2+^ entry that potentially results in an intracellular Ca^2+^ overload and produces the cardiac arrhythmogenecity following addition of FPL.

The cardiac action potential (AP) in ventricular myocytes is initiated by extracellular Na^+^ entry but subsequently involves Ca^2+^ entry, mainly through L-type voltage-dependent Ca^2+^ channels (LTCCs), that both maintains the cell depolarization and initiates excitation–contraction coupling. The latter process requires a Ca^2+^-induced Ca^2+^ release (CICR) through sarcoplasmic reticular (SR) ryanodine receptor (RyR2) Ca^2+^ release channels (Wehrens & Marks, 2003; Jiang *et al.* 2004; Scoote & Williams, 2004). The consequent rise in cytosolic [Ca^2+^] activates systolic cardiac muscle contraction. During diastole, the extra Ca^2+^ is removed from the cytoplasm through SR Ca^2+^-ATPase (SERCA2a) into the SR or extruded from the cell via the Na^+^–Ca^2+^ exchanger (NCX; Williams *et al.* 2001). Abnormalities in the Ca^2+^ homeostatic process have been implicated in triggered arrhythmias, such as those associated with the ventricular arrhythmogenesis leading to sudden cardiac death, particularly in cardiac failure and catecholaminergic polymorphic ventricular tachycardia (Pogwizd *et al.* 2001; Williams *et al.* 2001; Jiang *et al.* 2002; Wehrens & Marks, 2003; Balasubramaniam *et al.* 2005; Goddard *et al.* 2008). These arrhythmias may manifest as early after-depolarizations (EADs), delayed after-depolarizations (DADs) and action potential alternans.

The present experiments, performed in both whole hearts and single myocytes, explore the extent to which alterations in LTCC-mediated entry of Ca^2+^ might eventually trigger mechanisms of this kind, through the use of the specific L-type calcium channel modulator FPL-64716 (FPL; Baxter *et al.* 1993; Triggle 2004). FPL appears to prolong both the opening of L-type Ca^2+^ channels during depolarization and the time course of inactivation upon repolarization (Rampe & Lacerda, 1991; Zheng *et al.* 1991; Kunze & Rampe, 1992; Lauven *et al.* 1999; Fan *et al.* 2000). Patch clamp studies from rat ventricular myocytes showed that FPL increased the amplitude of both Ca^2+^ currents and Ca^2+^ transients more than it increased the rate of rise of Ca^2+^ transients. It also slowed activation and inactivation, and enhanced the duration of tail currents upon repolarization (Fan & Palade, 2002). Our findings indeed demonstrate such arrhythmogenic effects of FPL, attributable to an enhanced Ca^2+^ entry, probably accompanied by consequent alterations in SR Ca^2+^ store.

## Methods

### Animals

Mice bred against a 129 genetic background (Harlan UK Ltd, Bicester, UK), housed at room temperature, fed sterile rodent chow with free access to water at all times and subjected to 12 h–12 h light–dark cycles were studied at ages between 5 and 10 months. They were killed by cervical dislocation (Schedule 1, UK Animals (Scientific Procedures) Act 1986).

### Experiments on Langendorff-perfused hearts

Hearts were dissected out and immediately immersed into ice-cold bicarbonate-buffered Krebs–Henseleit (KH) buffer (containing, in mmol l^−1^): NaCl, 119; Na_2_CO_3_, 25; KCl, 4; KH_2_PO_4_, 1.2; MgCl_2_, 1.0; CaCl_2_, 1.8; glucose, 11; and sodium pyruvate, 2.0. The buffer was made daily and equilibrated with a 95% O_2_–5% CO_2_ gas mixture (British Oxygen Company, Manchester, UK) (Bethell *et al.* 1998). The electrophysiological experiments on arrhythmogenesis at the whole heart level used a Langendorff-perfused preparation adapted for the murine model (Papadatos *et al.* 2002; Balasubramaniam *et al.* 2003). The perfusate was passed through a water-jacket at a flow rate of 2.0–2.5 ml min^−1^ (model 505s, Watson-Marlow Berdel, Falmouth, UK). Fluid in the water-jacket was circulated with a circulator (model C−85A, Techne, Cambridge, UK) and warmed to 37°C by passing through a water-bath. The perfusate was then passed through 200 and 5 μm filters (Millipore, Watford, UK) before passing into the aorta of the heart. Hearts were thus perfused using buffer in which catecholamines were absent. They were then allowed to beat spontaneously until a steady state was reached, a time that permitted ample time for washout of any catecholamines that may have been present in the circulation at the time of killing.

The contracting heart was stimulated with a custom-made platinum stimulating electrode applied to the epicardial surface, usually for 15 min at 10 Hz, and was allowed to reach a physiologically steady state. The heart was paced from its right ventricle at three times its threshold level (between 3 and 5 V) using square-wave stimuli of 2 ms duration (Grass S48 stimulator, Grass Telefactor, Slough, UK). Monophasic action potentials (MAPs) were recorded from the epicardial basal surface of the left ventricle using a contact-type MAP electrode (Linton Instruments, Harvard Apparatus, Edenbridge, UK).

Following the equilibration period, hearts were first studied in conditions of spontaneous activity, which typically took place at a heart rate of ∼6 Hz. They were then subjected to constant ventricular pacing at cycle lengths of 8 and 10 Hz. Finally, they were subjected to programmed electrical stimulation (PES) using a variation of existing clinical techniques (Saumarez & Grace, 2000; Saumarez *et al.* 2003). The stimulation sequence consisted of a drive train of pacing stimuli (S1) applied with a 200 ms cycle length, with an extra stimulus (S2) inserted every eighth beat performed at cycle lengths of 8 and 10 Hz for determination of the ventricular effective refractory period (VERP).

The MAP signals were amplified, filtered (band-pass filter 0.5 Hz to 1 kHz, Gould-Nicolet Technologies, Ilford, UK) and digitized at a frequency of 5 kHz using an analog-to-digital converter (model 1401plus, Cambridge Electronic Design, Cambridge, UK). Monophasic APs that were considered for analysis were all stable for more than 30 min, and the data were manually reviewed using Spike2 software (CED, Cambridge, UK). According to accepted criteria regarding MAP morphology, MAP traces selected should have a stable baseline with a rapid upstroke without inflection or negative spikes, consistent amplitude >1 mV, and rapid first phase repolarization lacking an early plateau. Action potential duration (APD) was analysed at 30, 50, 70 and 90% repolarization (APD_30_, APD_50_, APD_70_ and APD_90_) following previous MAP analysis protocols (Gussak & Antzelevitch, 2000; Fabritz *et al.* 2003). Results are all expressed as means ±s.e.m.

Pharmacological agents studied were all purchased from Sigma-Aldrich, Poole, UK. The Ca^2+^ channel agonist FPL-64716 (FPL) was initially made up with dimethyl sulphoxide (DMSO) to make a stock concentration of 5 mm. Stock solutions of FPL were wrapped in foil because they are photosensitive and stored at −20°C. Cyclopiazonic acid (CPA) was dissolved in 96% ethanol to make an initial stock solution of 10 mm, which was stored at −20°C. Diltiazem was dissolved in distilled water to make a stock concentration of 1 mm and kept refrigerated at 4°C. Final drug solutions used were diluted with KH buffer made fresh before every experiment. The FPL was diluted to a working concentration of 1 μm. The CPA was diluted to an intermediary solution of 10 μm and then further diluted to a concentration of 150 nm. Nifedipine and diltiazem were both used at a final concentration of 100 nm.

Arrhythmogenic phenotypes in the form of after-depolarizations, delayed after-depolarizations, alternans, and monomorphic and polymorphic ventricular tachycardia were examined both before and following introduction of FPL in both control conditions and following subsequent additions of nifedipine, diltiazem and CPA. After-depolarizations were defined as depolarizing electrical events that interrupted the restitution phases of action potentials, whereas premature activations took place following full action potential recovery. Spontaneous tachycardias prolonged over more than 30 s were defined as sustained, whereas those lasting less than 30 s were defined as non-sustained.

Statistical analysis of results obtained in the different pharmacological conditions was carried out using either paired or unpaired two-way ANOVA (SPSS Inc., Chicago, IL, USA) for continuous data and Fisher's exact test for categorical data. Results are all expressed as means ±s.e.m., and differences were considered significant at a level of *P* < 0.05.

### Measurements of myocyte [Ca^2+^]

Single-cell experiments used mouse ventricular myocytes isolated by adapting an established enzymatic digestion described previously (Harding *et al.* 1992). Following killing, hearts were excised rapidly and cannulated before being connected to a Langendorff perfusion system. The heart was then perfused for 1.5 min at 3 ml min^−1^ with perfusion buffer containing (mm): NaCl, 120; MgSO_4_, 5; KCl, 5.4; sodium pyruvate, 5; Hepes, 10; glucose, 5; taurine, 20; nitrilotriacetic acid (NTA), 5 (Sigma-Aldrich); and adjusted using NaOH to pH 7.0 at 37°C. Subsequently, the heart was perfused for 25–28 min at 3 ml min^−1^ with digestion buffer containing (mm): NaCl, 120; MgSO_4_, 5; KCl, 5.4; sodium pyruvate, 5; Hepes, 10; glucose, 5; and taurine, 20, to which was added collagenase type II (Worthington, Lakewood, NJ, USA; final concentration 1 mg ml^−1^), hyaluronidase (Sigma-Aldrich; final concentration 1 mg ml^−1^) and 200 μm Ca^2+^ and adjusted using NaOH to pH 6.8 at a temperature of 37°C. The pale and swollen heart was then removed and cut below the atria. The digested tissue was teased gently with fine forceps, and the dissociated cells were transferred to a conical tube containing 12.5 ml digestion buffer with 50 mg bovine serum albumin (BSA) to inactivate digestion. The resulting myocytes were subjected to further trituration using sterile plastic transfer pipettes, and allowed to sediment by gravity for 10 min.

The cell suspension was then centrifuged for 1.5 min at 100 ***g*** and the pellets resuspended twice in wash buffer containing (mm): NaCl, 119; KCl, 4.2; MgSO_4_, 0.94; KH_2_PO_4_, 1.2; Hepes, 20; glucose, 11.5; taurine, 20; and 1 mg ml^−1^ BSA, and bubbled with 95% O_2_–5% CO_2_, pH 7.4, to remove all traces of NTA, collagenase and hyaluronidase. Calcium chloride was then cautiously reintroduced by stepwise (0.2 mm) additions to reach an approximate concentration of 1.25 mm, by centrifuging the preparation at each Ca^2+^ reloading stage to remove dead or swollen cells. During this process, myocytes were examined periodically under the microscope to confirm a good yield of rod-shaped myocytes (better than 60%) before continuing the experiments. Cells were then transferred into a conical tube (BD Biosciences, Oxford, UK) and incubated at room temperature. The isolated myocytes were then placed in a control Hepes-buffered Krebs–Henseleit solution that was the same as the wash buffer mentioned previously without BSA and had a pH of 7.2, and transferred to a Perspex chamber 10 mm × 4 mm × 6.25 mm (length × width × depth). The myocytes spontaneously attached to the glass coverslip (3 cm × 3 cm, grade 1 coverslip (Merck, Hoddesdon, Herts, UK) forming the floor of the chamber. The myocytes were stimulated to contract using two in-built platinum field electrodes running the length of the chamber through which the periodic field stimulation was applied using a custom-built square-wave stimulator. This applied successive 40–60 V steps of duration 2.2 ms, immediately followed by a step of similar duration and amplitude but opposite polarity at a pacing frequency of 0.5 Hz. During this stimulation, cells were loaded with the acetoxymethyl (AM) ester of fluo-3 (Invitrogen, Paisley, UK; 50 μg vial diluted in 30 μl pluronic acid to give a stock concentration of 1.476 mm) by incubating the cells in a bath of volume 500 μl containing KH buffer (with 1.25 mm Ca^2+^) with 2 μl of fluo−3 AM solution for 15 min at room temperature in the dark. The bath with the myocytes was then transferred onto the stage of a Zeiss LSM-510 laser scanning confocal microscope system (Carl Zeiss Ltd, Welwyn Garden City, UK) with a ×20 air objective (NA 0.5; confocal aperture 1000 μm, slice thickness <42.4 μm) on a Zeiss Axiovert 100 M inverted microscope. Fluo−3 fluorescence emission was excited with a 488 nm argon laser and measured at 505–550 nm. Images were analysed using an in-house custom-written program. A series of 500 frames sampled at 98 ms per frame (128 × 128 pixels per frame) were used initially to monitor fluorescence changes over time. The appropriateness of the chosen frame capture rate was corroborated in some experiments by collecting data in the faster line-scan mode with a sampling rate of 960 μs per line. All traces had stable baselines, confirming consistent laser intensities and detector gains. The peak normalized fluorescence (*F*/*F*_0_) values following each response to each stimulus were calculated for each time series, and a mean was acquired for each series. Moreover, the mean peak *F*/*F*_0_ values as well as the baseline diastolic values were calculated. The results are expressed as mean values ±s.e.m. and compared using ANOVA (SPSS Inc.). Different solutions were perfused through the perfusion chamber as required. All confocal microscope studies were carried out at room temperature.

## Results

### Recordings of MAPs demonstrate arrhythmic effects of FPL

**Figure 1:**
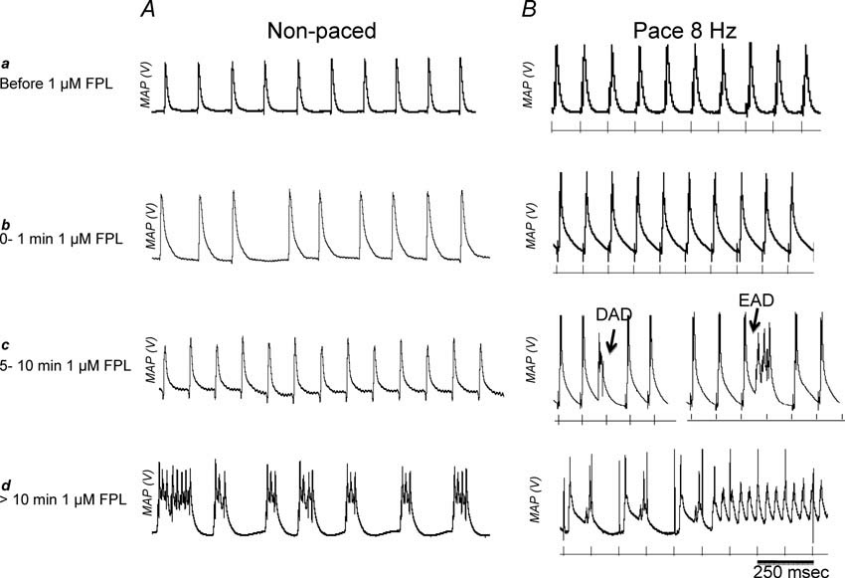
Examples of monophasic action waveforms Epicardial monophasic action waveforms from epicardial surfaces of the left ventricles of Langendorff-perfused hearts during both non-paced activity (*A*) and regular pacing at 8 Hz (*B*) before (*a*) and at different times following the introduction of 1 μm FPL-64716 (FPL) into the perfusing KH buffer solution (*b–d*).

Previous work has demonstrated that FPL (1 μm) increases extracellular Ca^2+^ entry through L-type Ca^2+^ channels, thereby enhancing cytosolic Ca^2+^ loading (Fan & Palade, 2002). It therefore served as a useful pharmacological tool to assess the effect of the resulting alterations of cellular Ca^2+^ homeostasis on cardiac arrhythmogenic properties. Such experiments first compared MAP waveforms from epicardial surfaces of the left ventricles of eight Langendorff-perfused hearts during both spontaneous activity (Fig. 1*A*) and during regular pacing at 8 Hz (Fig. 1*B*) before and at different times following the introduction of FPL (1 μm) into the perfusing KH buffer solution. The traces shown in each panel exemplify results from ∼2 min of recordings in each condition. Before introduction of FPL all hearts showed normal MAP waveforms whose time courses resembled those reported on earlier occasions (Fabritz *et al.* 2003; Killeen *et al.* 2007*a*) and no evidence of arrhythmogenic behaviour (Fig. 1*Aa* and *Ba*).

**Figure 2:**
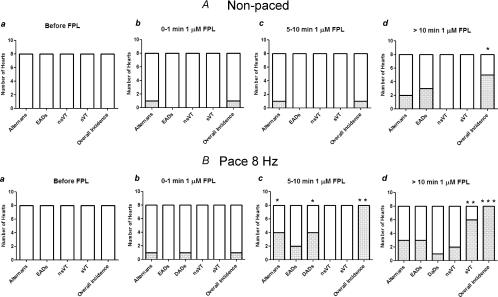
Arrhythmic phenomena in non-paced and paced hearts Numbers of hearts showing arrhythmic phenomena out of a total of *n*= 8 studied during both non-paced activity (*A*) and regular pacing at 8 Hz (*B*) before (*a*) and at different times following the introduction of 1 μm FPL-64716 (FPL) into the perfusing KH buffer solution (*b–d*). The bar graphs show incidences of hearts having one or more episodes of alternans (*Ab–Ad* and *Bb–Bd*), early and delayed after-depolarizations (EADs and DADs; *Ad*, *Bc* and *Bd*) or episodes of ventricular tachycardia (VT; *Bd*). The bar graphs also show results of Fisher's exact tests for these incidences when compared with control values obtained in the absence of FPL (*P < 0.01, **P < 0.001 and ***P < 0.0001; open bars indicate and absence or insignificant incidence of the arrhythmic phenomenon).

Figure 2 summarizes numbers of hearts showing each phenomenon, as well as the results of Fisher exact tests for these incidences when compared with control values obtained in the absence of FPL. Introduction of FPL then led to a progressive development, over 0–10 min, of a variety of waveform abnormalities in both non-paced and paced hearts (Figs 1*Ab–d* and *Bb–d* and 2*Ab–d* and *Bb–d*). During spontaneous activity, 1 min of exposure to FPL left MAP waveforms and rhythm relatively normal apart from a single episode of 40 pairs of action potentials showing a stable interval alternans, attributable to either ventricular or supraventricular changes, in a single heart (Figs 1*Ab* and 2*Ab*). At between 5–10 min of FPL perfusion, a single heart showed 120 pairs of action potentials in alternans (Figs 1*Ac* and 2*Ac*). At >10 min following introduction of FPL, two hearts showed 12 and 50 pairs of APs in alternans, and MAPs in three hearts showed repetitive spikes interrupting the time courses of the AP recoveries (Fig. 1*Ad*), resulting in a statistically significant (*P*= 0.009) incidence of arrhythmic events compared with control results observed before addition of FPL (Fig. 2*Ad*).

In paced hearts, with 1 min of exposure to FPL there were no arrhythmic episodes (Fig. 1*Bb*) apart from a single 40 s episode of stable alternans and one DAD in one heart (Fig. 2*Bb*). A total of four hearts perfused for 5–10 min with FPL exhibited episodes of alternans with a mean duration of 25 ± 7.6 s (*P*= 0.07, compared with control); of these, two hearts showed EADs that intercepted the recovery phase of approximately two and six out of 20 successive APs. Moreover, four hearts showed DADs, giving an overall statistically significant incidence of arrhythmic phenomena (*P*= 0.006) compared with control hearts (Figs 1*Bc* and 2*Bc*). With >10 min exposure to FPL, three hearts showed five episodes of stable alternans of mean duration 21 ± 6.4 s. In addition, three hearts showed EADs that interrupted six, 10 and three out of 20 successive action potentials, three hearts showed DADs, two hearts showed episodes of ventricular tachycardia (VT) lasting <30 s, and six hearts showed episodes of VT lasting >30 s (Figs 1*Bd* and 2*Bd*). The incidence of sustained VT (sVT) and the overall incidence of arrhythmic events were both significantly higher (*P*= 0.006, *P*= 0.0001) than corresponding incidences obtained before addition of FPL.

**Figure 3:**
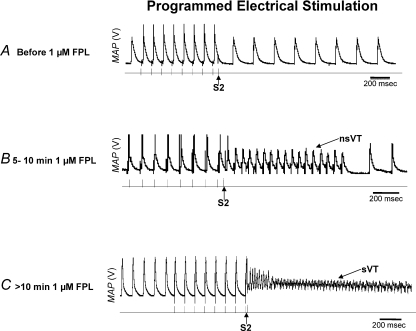
Monophasic action potentials recorded from the epicardium of hearts in the absence and presence of FPL Monophasic action potentials recorded from the epicardium of hearts before (*A*), 5–10 min (*B*) and >10 min after the addition of 1 μm FPL (*C*). All experiments were performed during PES at 8 Hz; the arrows indicate S2 extra-stimuli. The examples illustrated that before addition of FPL (*A*), isolated perfused hearts showed a persistently regular rhythm with no arrhythmogenic events during the PES procedures. The S2 extra-stimuli initiated an episode of nsVT lasting for less than 30 s in 7 out of 8 hearts perfused with FPL for 5–10 min (*B*). All 8 hearts showed episodes of sVT lasting for more than 30 s with further perfusion with FPL for more than 10 min (*C*).

Programmed electrical stimulation procedures then provided quantifiable tests for arrhythmic substrate as employed on previous experimental and clinical occasions (Saumarez & Grace, 2000; Balasubramaniam *et al.* 2003). Figure 3 illustrates findings showing that FPL treatment resulted in significant arrhythmic substrate. Thus: (1) regularly paced untreated hearts showed no arrhythmic incidents (*n*= 8; Fig. 3*A*); (2) seven out of eight hearts perfused with FPL for 5–10 min showed non-sustained VT (nsVT), representing a significant increase in such arrhythmic incidence (Fig. 3*B*; Fisher exact test giving a two-tailed probability of *P* < 0.001); and (3) all eight hearts showed episodes of sVT with further perfusion with FPL for more than 10 min (Fig. 3*C*; *P* < 0.0001).

### Pharmacological modifications of the effects of FPL

**Figure 4:**
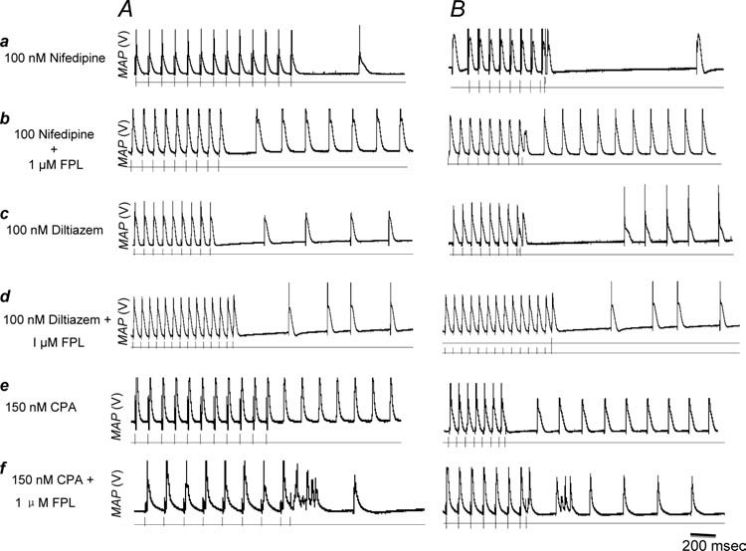
Epicardial monophasic action potential recordings in paced hearts and in hearts undergoing PES in the presence of various drugs Epicardial MAP recordings obtained from both regularly paced hearts (*A*) and hearts undergoing PES (*B*) pretreated with 100 nm nifedipine (*a*), 100 nm diltiazem (*c*) and 150 nm CPA (*e*) before restoring 1 μm FPL to the buffer. None of the hearts showed arrhythmic phenomena following pretreatment (*a*, *c* and *e*). Inclusion of either nifedipine or diltiazem during subsequent FPL treatment totally suppressed FPL-induced VT (*b* and *d*, respectively) through either pacing protocol. However, 150 nm CPA in combination with a subsequent addition of 1 μm FPL (*f*) abolished FPL-induced VT although it did permit EAD episodes following the shortest S2 stimuli in each of *n*= 4 hearts.

We next explored the effects of pretreatments with the dihydropyridine Ca^2+^ channel blocker nifedipine (100 nm), the benzothiazepine Ca^2+^ blocker diltiazem (100 nm) and the effects of the SERCA inhibitor CPA (150 nm) before restoring FPL to the buffer, in both regularly paced hearts (Fig. 4*A*) and in hearts undergoing PES (Fig. 4*B*). Cyclopiazonic acid (Goeger *et al.* 1988; Mason *et al.* 1989; Seidler *et al.* 1989) specifically inhibits SERCA activity in myocardial vesicle preparations by ∼50% at 100–200 nm and completely at 10 μm (Schwinger *et al.* 1997) whilst sparing Ca^2+^ sensitivity in the contractile myofilaments (Takahashi *et al.* 1995), Ca^2+^ currents (Bonnet *et al.* 1994) and NCX activity (Yard *et al.* 1994).

None of the hearts on pretreatment with nifedipine (*n*= 4 hearts; Fig. 4*Aa* and *Ba*), diltiazem (*n*= 4 hearts; Fig. 4*Ac* and *Bc*) or CPA (*n*= 4; Fig. 4*Ae* and *Be*) showed arrhythmic phenomena. Inclusion of either nifedipine or diltiazem during subsequent FPL treatment totally suppressed FPL-induced VT (Fig. 4*Ab*, *Ad*, *Bb* and *Bd*) through either pacing protocol in the same hearts. Treatment with CPA in combination with a subsequent addition of FPL (Fig. 4*Af* and *Bf*) abolished FPL-induced VT, although it did permit EAD episodes following the shortest S2 stimuli in all four hearts (Fisher's exact test, *P*= 0.002 when tested against results with FPL alone in each case).

### Correlations between arrhythmic actions and AP waveform

Exposure to FPL thus produced VT in isolated murine hearts, and this occurred along with phenomena associated with triggered arrhythmogenesis such as EADs and alternans. The experiments that followed investigated the extent to which FPL produces changes in APD and refractory period, since alterations in these have been observed in previous long QT syndrome (LQTS) and hypokalaemic murine cardiac models and have been implicated in the re-entrant substrate responsible for their abnormal electrical activity (Stokoe *et al.* 2007*a*,*b*; Thomas *et al.* 2007*b*). Both mechanisms could co-exist. Timothy syndrome, attributed to abnormalities in the LTCC, results in a multisystem disorder that also causes LQTS (Splawski *et al.* 2004, 2005).

Firstly, epicardial APD values were accordingly measured at *x*= 30, 50, 70 and 90% recovery (APD_30_, APD_50_, APD_70_ and APD_90_) from left ventricular MAPs obtained from hearts paced at 8 Hz (Table 1). In control hearts, APD_90_ was 48 ± 3.5 ms (*n*= 6 hearts), in agreement with previous reports (e.g. Killeen *et al.* 2007*b*). We then investigated the effects of FPL (1 μm; *n*= 6 hearts), at different perfusion times (0–1, 5–10 and >10 min) identical to those adopted above upon values of APD*_x_*. Application of two-way ANOVA to each of the above groups demonstrated that there were no significant differences between treated or untreated groups with the exception of APD_30_ values obtained between 5–10 min. Therefore, FPL did not significantly alter action potential time course despite the occurrence of EADs and alternans but not sVT at times <10 min and the appearance of all three phenomena at times >10 min. This progressive development of arrhythmogenesis is therefore more likely to arise from progressive increases in intracellular Ca^2+^ resulting from increased net Ca^2+^ influx brought about by FPL. The findings complement recent reports in a murine RyR2 model whose arrhythmogenic properties were attributable to alterations in RyR2 (Goddard *et al.* 2008). They contrast with re-entrant arrhythmogenic properties associated with altered action potential kinetics demonstrated in models for long QT syndrome type 3 (LQT3) (Thomas *et al.* 2007*a*).

**Table 1:** Left ventricular epicardial action potential durations at different repolarization times in regularly paced (8 Hz) hearts at different perfusion times in the presence and absence of 1 μm FPL-64716

	APD_30_ (ms)		APD_50_ (ms)		APD_70_ (ms)		APD_90_ (ms)	
	Mean	s.e.m.	Mean	s.e.m.	Mean	s.e.m.	Mean	s.e.m.
KH buffer (*n*= 6)	6	1.0	14	1.3	27	3.0	48	3.5
0–1 min KH buffer (*n*= 6)	6	1.4	13	3.1	24	4.0	47	3.0
5–10 min KH buffer (*n*= 6)	4	0.5	14	1.5	29	3.6	54	2.3
>10 min KH buffer (*n*= 6)	6	0.6	13	2.8	27	3.6	54	1.5
KH buffer (*n*= 6)	11	0.1	18	0.1	26	0.2	43	0.2
0–1 min 1 μm FPL (*n*= 6)	10	1.3	20	1.5	31	1.7	49	1.2
5–10 min 1 μm FPL (*n*= 6)	13	4.1	22	5.0	33	4.2	53	1.5
>10 min 1 μm FPL (*n*= 6)	6	2.2	11	4.2	22	4.5	60	2.8

*n* gives number of hearts.

**Figure 5:**
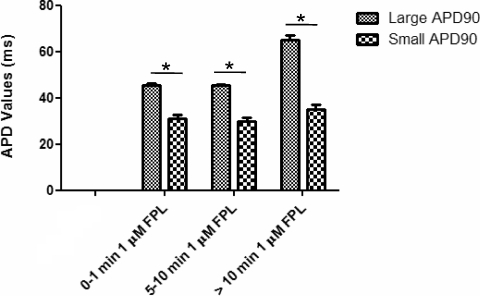
The effect of alternans on APD_90_ values The APD_90_ values in alternans were calculated for alternans that appeared 1 min following introduction of FPL. However, the presence of alternans significantly (*P < 0.001) decreased APD_90_ values with increased FPL perfusion times after 1 min, 5–10 min and >10 min perfusion with FPL.

Secondly, we followed APD_90_ values for successive alternate MAPs during episodes of alternans in paced hearts. At 1, 5–10 and >10 min following FPL introduction, the mean duration of the shorter of each pair of MAPs was 31 ± 1.5 (*n*= 1 heart, 20 action potentials), 30 ± 1.5 (*n*= 4 hearts, 20 action potentials) and 35 ± 1.8 ms (*n*= 3 hearts); the corresponding value for the longer of each pair was 45 ± 0.7 (*n*= 1 heart), 46 ± 0.3 (*n*= 4 hearts) and 65 ± 2.1 ms (*n*= 3 hearts), respectively (Fig. 5).

Thirdly, neither pretreatment with nifedipine (*n*= 6 hearts), diltiazem (*n*= 5 hearts) and CPA (*n*= 9 hearts) nor a further addition of FPL resulted in any significant change in APD*_x_* as indicated by ANOVA (Table 2).

**Table 2:** Effects of nifedipine, diltiazem and cyclopiazonic acid on action potential duration in the presence and absence of 1 μm FPL-64716

	APD_30_ (ms)		APD_50_ (ms)		APD_70_ (ms)		APD_90_ (ms)	
	Mean	s.e.m.	Mean	s.e.m.	Mean	s.e.m.	Mean	s.e.m.
KH buffer (*n*= 4)	10	2.0	19	3.1	30	3.2	49	1.7
100 nm nifedipine (*n*= 6)	6	1.7	19	1.8	30	1.6	49	1.4
100 nm nifedipine + 1 μm FPL (*n*= 8)	9	1.4	17	1.6	30	1.5	49	1.1
KH buffer (*n*= 5)	7	1.5	16	2.2	30	2.5	52	2.5
100 nm diltiazem (*n*= 5)	4	0.6	13	1.0	28	1.9	50	2.1
100 nm diltiazem + 1 μm FPL (*n*= 7)	6	0.6	16	1.5	30	1.5	53	0.9
KH buffer (*n*= 5)	11	1.5	20	1.5	30	1.4	47	1.4
150 nm CPA (*n*= 9)	13	1.6	20	2.3	30	2.9	49	3.2
150 nm CPA + 1 μm FPL (*n*= 7)	10	1.9	19	2.1	29	2.9	47	3.7

*n* gives number of hearts.

### Calcium transients in single mouse myocytes

**Figure 6:**
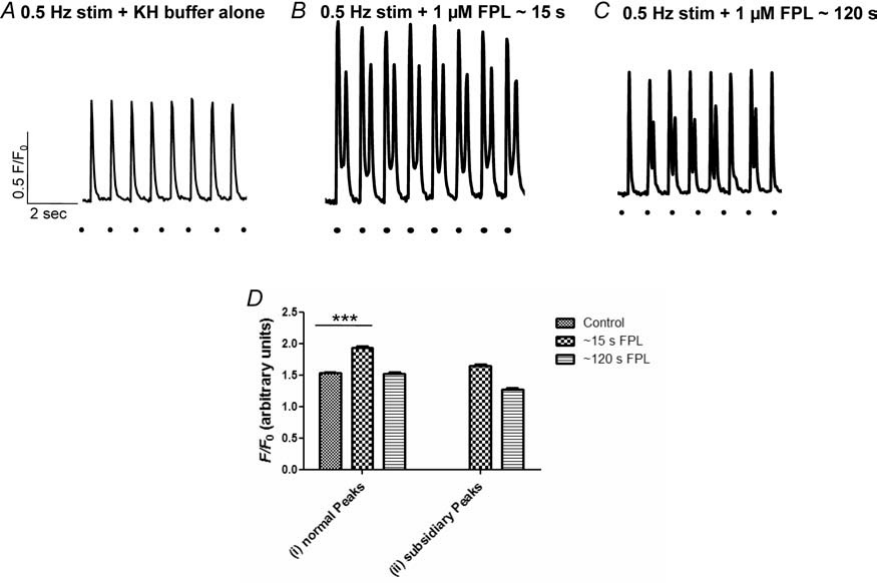
Fluo−3 fluorescence measurements in isolated myocytes studied using confocal microscopy Normalized fluorescence (*F*/*F*_0_) plotted against time with myocyte exposed to periodic field stimulation in perfusion buffer alone prior to any pharmacological manoeuvre (*A*) and records obtained following addition of 1 μm FPL to the buffer for approximately 15 s (*B*) and approximately 120 s (*C*). *D* shows overall mean peak *F*/*F*_0_ values taken from the entire set of myocytes. The overall mean peak *F*/*F*_0_ values are represented for the entire set of myocytes (*Di*) and subsidiary events (*Dii*).

**Figure 7:**
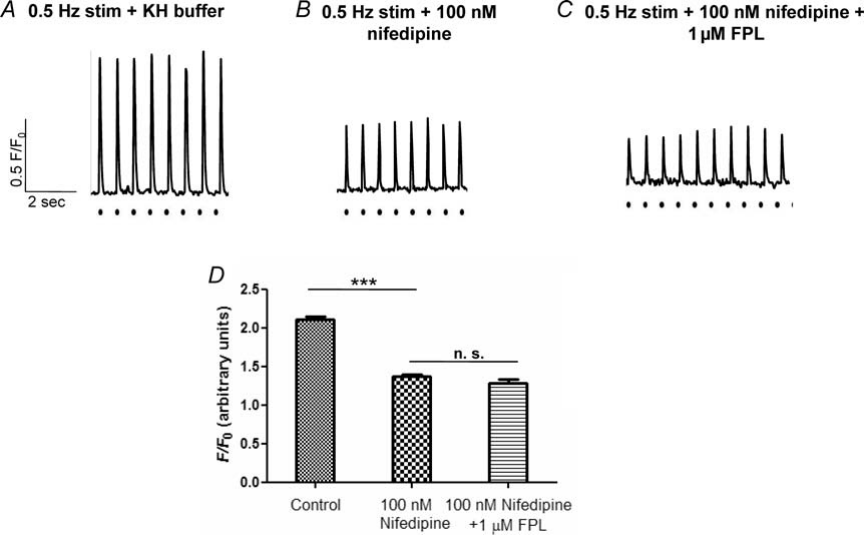
Calcium transients in regularly stimulated isolated myocytes in the presence of nifedipine and FPL Calcium transients in regularly stimulated (0.5 Hz) isolated myocytes before (*A*) and after addition of 100 nm nifedipine (*B*) and a further addition of FPL (*C*). *D*, nifedipine significantly (***P < 0.05; one-way ANOVA) reduced peak *F*/*F*_0_ from 2.11 ± 0.04 to 1.37 ± 0.02 (*n*= 9 cells), but there were no further changes following introduction of FPL, which left a peak *F*/*F*_0_ of 1.28 ± 0.05 (*n*= 6 cells).

**Figure 8:**
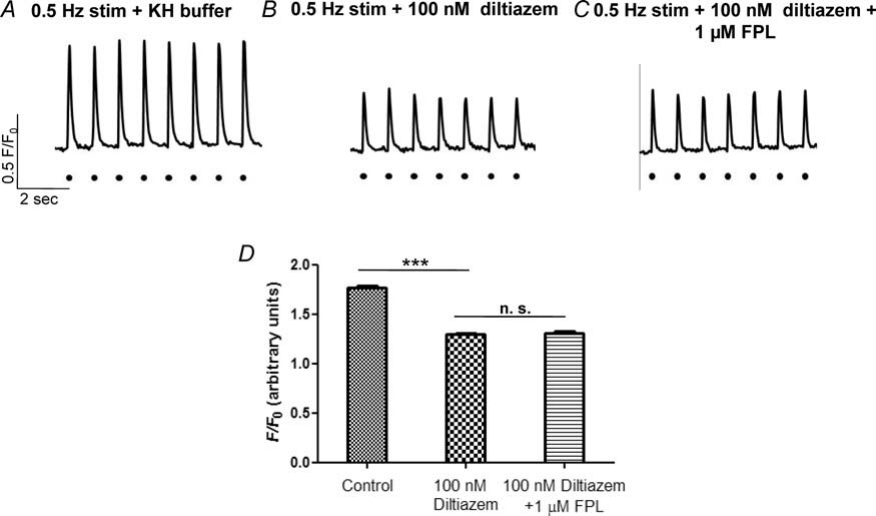
Calcium transients in regularly stimulated isolated myocytes in the presence of diltiazem and FPL Calcium transients in regularly stimulated (0.5 Hz) isolated myocytes before (*A*) and after addition of 100 nm diltiazem (*B*) and a further addition of FPL (*C*). *D*, diltiazem significantly (***P < 0.05; one-way ANOVA) reduced peak *F*/*F*_0_ from 1.77 ± 0.02 (*n*= 3 cells) to 1.30 ± 0.01 (*n*= 3 cells), but there were no further changes following introduction of FPL, which left a peak *F*/*F*_0_ of 1.31 ± 0.01 (*n*= 4 cells).

**Figure 9:**
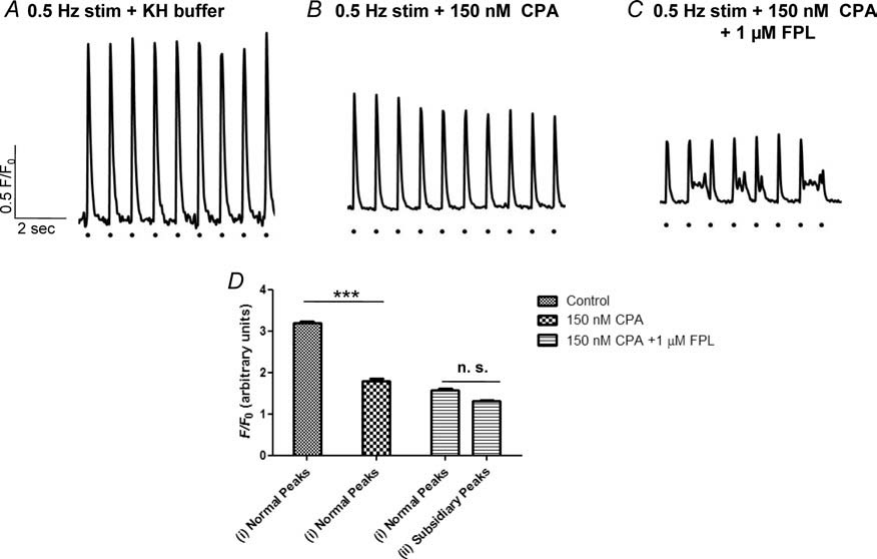
Calcium transients in regularly stimulated isolated myocytes in the presence of CPA and FPL Calcium transients in regularly stimulated (0.5 Hz) isolated myocytes before (*A*) and after addition of 150 nm CPA (*B*) and a further addition of FPL (*C*). *D*, CPA significantly (***P < 0.05; one-way ANOVA) reduced peak *F*/*F*_0_ from 3.20 ± 0.04 (*n*= 9 cells) to 1.80 ± 0.06 (*n*= 12 cells), but there were no further changes in peak *F*/*F*_0_ following introduction of FPL, which left a peak *F*/*F*_0_ of 1.57 ± 0.05 (*n*= 6 cells), but an appearance of persistent spontaneous ectopic Ca^2+^ peaks in the intervals between stimuli whose peak *F*/*F*_0_ was 1.32 ± 0.02 (*n*= 6 cells; *C* and *D*).

The experiments that followed correlated these arrhythmic effects with changes in the amplitude and pattern of Ca^2+^ transients measured in fluo-3 loaded isolated murine cardiac myocytes. These were subjected to regular stimulation (0.5 Hz), whose timing is indicated by the dots below the *F*/*F*_0_ traces in Figs 6–9. Cells were studied in KH buffer under identical FPL concentrations, in the presence and absence of pretreatments with nifedipine, diltiazem and CPA. In all these experimental conditions, *F*/*F*_0_ traces showed stable baselines and reproducible peak responses to regular stimulation with minimal evidence of fluorophor bleaching over the adopted sampling periods as assessed by readings obtained at the beginning and end of the adopted, 50 s, sampling periods (*P*≫ 5%).

Before addition of FPL, the Ca^2+^ signals observed immediately following commencement of stimulation consisted of series of responses with consistent amplitudes, each directly following the individual stimuli with a peak *F*/*F*_0_ of 1.53 ± 0.02 (events counted over a standard sampling period of 50 s; *n*= 6 cells; cells from at least 2 hearts studied in each condition). The myocytes did not show spontaneous ectopic peaks in the intervals between stimuli, nor did they show any subsidiary events during the recovery phases of the evoked Ca^2+^ transients (Fig. 6*A*).

In contrast, resumption of scanning within 15 s of addition of FPL demonstrated *F*/*F*_0_ transients with increased amplitudes of 1.93 ± 0.03 (*n*= 3 cells, *P* < 0.001) that showed subsidiary events (1.64 ± 0.03, *n*= 6 cells, 2 hearts; Fig. 6*B*). The additional Ca^2+^ entry from either the extracellular space or Ca^2+^ stores reflected in such subsidiary events would lead to a progressive cytosolic Ca^2+^ loading, which, if the cause of the observed arrhythmogenesis, would account for the gradual onset of the arrhythmic phenomena described here. However, following this initial scanning, with continued stimulation for >120 s, resumption of scanning demonstrated *F*/*F*_0_ transients whose amplitudes were significantly lower (peak *F*/*F*_0_ of 1.53 ± 0.02, *n*= 6 cells, 2 hearts, *P* < 0.05) with subsidiary events of amplitude 1.27 ± 0.02 (*n*= 3 cells; Fig. 6*C* and *D*), a finding compatible either with photobleaching or a consequent depletion of the Ca^2+^ store.

The effects of the pharmacological agents nifedipine (100 nm; Fig. 7), diltiazem (100 nm; Fig. 8) and CPA (150 nm; Fig. 9) were then compared before and 10 min after a subsequent addition of FPL in similarly stimulated cells. Despite their sharply differing sites of action in influencing Ca^2+^ homeostasis, such manoeuvres yielded similar results. Thus, the addition of nifedipine, diltiazem and CPA all reduced peak *F*/*F*_0_ in a similar manner, significantly (*P* < 0.05; one-way ANOVA), from 2.11 ± 0.04 (*n*= 6 cells), 1.77 ± 0.02 (*n*= 3 cells) and 3.20 ± 0.04 (*n*= 9 cells; Figs 7*A* and *D*, 8*A* and *D* and 9*A* and *D*) to 1.37 ± 0.02 (*n*= 9 cells), 1.30 ± 0.01 (*n*= 3 cells) and 1.80 ± 0.06 (*n*= 12 cells) (Figs 7*B* and *D*, 8*B* and *D* and 9*B* and *D*), respectively. These findings agree with earlier results that had used similar concentrations of nifedipine and diltiazem (Balasubramaniam *et al.* 2004).

However, in all three cases, following washout and reintroduction of solutions that also included 1 μm FPL, giving an overall sequence of solution changes identical to that used in the corresponding experiments on whole hearts, there was no further significant change in values of *F*/*F*_0_, which were left at 1.28 ± 0.05 (*n*= 6 cells), 1.31 ± 0.01 (*n*= 4 cells) and 1.57 ± 0.05 (*n*= 6 cells; Figs 7*C* and *D*, 8*C* and *D* and 9*C* and *D*, respectively) even after 1 min following perfusion of the new agents. Furthermore, the presence of nifedipine and diltiazem abolished both ectopic and subsidiary Ca^2+^ release events (Figs 7*C* and *D* and 8*C* and *D*) in parallel with the similar absence of any arrhythmic phenomena in the whole hearts. In contrast, myocytes treated with both CPA and FPL showed persistent spontaneous ectopic Ca^2+^ peaks in the intervals between stimuli. There were also subsidiary events whose peak *F*/*F*_0_ (1.32 ± 0.02; *n*= 6 cells) was similar to those observed with FPL. Nevertheless they occurred significantly less frequently, in 5 ± 0.20 out of 23 evoked transients (*n*= 22 cells, 4 hearts; Fig. 9*C*) than in myocytes treated with FPL alone. These last findings correlate with the persistence of transient arrhythmic events observed in the whole hearts.

## Discussion

The present experiments explored the extent to which alterations in LTCC-mediated entry of Ca^2+^ might eventually trigger Ca^2+^-mediated arrhythmogenesis in isolated perfused murine hearts through use of the specific L-type calcium channel modulator FPL-64716 (Baxter *et al.* 1993; Triggle, 2004). FPL is known to increase the contractility of both smooth and cardiac muscle (Zheng *et al.* 1991; Rampe & Dage, 1992; Rampe *et al.* 1993). Murine hearts have already successfully been used to replicate arrhythmogenic properties associated with Brugada and LQT3 syndromes in which re-entrant mechanisms have been implicated (Stokoe *et al.* 2007*a*,*b*; Thomas *et al.* 2007*a*,*b*). Recently, murine models have been used in studies of an arrhythmogenic condition related to abnormal RyR2 function (Cerrone *et al.* 2005; Goddard *et al.* 2008). Their use in the present study permitted our examination not only of the physiological effects of alterations in Ca^2+^ homeostasis on murine cardiac function, but also their comparison with these previously described murine models.

Treatment with FPL resulted in a gradual development of arrhythmogenic phenomena in an absence of marked alterations in action potential waveform. This would be unexpected had FPL acted as a simple Ca^2+^ channel agonist to increase inward Ca^2+^ current. However, FPL is likely to have more complex actions in additionally slowing L-type Ca^2+^ channel opening during depolarization and slowing inactivation upon repolarization (Rampe & Lacerda, 1991; Zheng *et al.* 1991; Kunze & Rampe, 1992; Lauven *et al.* 1999; Fan *et al.* 2000; Fan & Palade, 2002), features that would compromise its full agonist action, and any consequence upon subsequent NCX activity (see e.g. Noble *et al.* 2007) in the situation of the relatively brief, triangulated action potentials (Danik *et al.* 2002; Hondeghem, 2007; Milberg *et al.* 2007) found in murine systems.

In the Langendorff-perfused murine hearts, introduction of FPL resulted in a gradual development (over 10 min) of diastolic electrical events and alternans in non-paced hearts. In paced hearts, FPL led to an additional appearance of arrhythmic, nsVT and sVT phenomena. Finally, hearts were subjected to PES. Results from this have been shown to correlate with arrhythmogenic tendency in genetically modified hearts modelling Brugada syndrome (Stokoe *et al.* 2007*a*), LQT3 (Head *et al.* 2005; Stokoe *et al.* 2007*b*) and long QT syndrome type 5 (LQT5) (Balasubramaniam *et al.* 2003; Thomas *et al.* 2007*b*) and to provide indications of clinical arrhythmogenic risk (Saumarez & Grace, 2000; Saumarez *et al.* 2003; Turner *et al.* 2005). In the present experiments, this procedure resulted in nsVT and sVT after 5–10 and >10 min perfusion, respectively. However, there were no accompanying alterations in APD when values obtained at matched times were compared in the presence and absence of FPL. This contrasts with the increases in APD associated with re-entrant substrate in other murine models (Killeen *et al.* 2007*a*).

In contrast, diastolic events of the kind observed here have previously been attributed to a range of Ca^2+^ homeostatic events. Thus, EADs have been attributed to L-type Ca^2+^ current (*I*_Ca_) reactivation during prolonged AP plateaus (Zaugg & Buser, 2001; Bers, 2002*a*,*b*, 2008; Roden, 2003; Laurita & Katra, 2005) and DADs may follow spontaneous release of intracellularly stored SR Ca^2+^, leading to cytosolic Ca^2+^ waves and oscillations and increased NCX activity (Kass & Tsien, 1982; Orchard *et al.* 1983; Stern *et al.* 1983; Wier *et al.* 1983; Marban *et al.* 1986; January *et al.* 1988).

The alternans that involved APD parallel similar phenomena in clinical situations, in which such beat-to-beat fluctuations in the electrocardiographic T-wave are thought to be a strong diagnostic precursor of ventricular tachycardias and ventricular fibrillation, potentially leading to sudden cardiac death (Lee *et al.* 1987, 1988; Bloomfield *et al.* 2006). They have also been observed and related to arrhythmogenic tendency in hypokalaemic murine models at high heart rates (short baseline cycle lengths; Sabir *et al.* 2008*b*). Previous studies had attributed alternans to fluctuations in cytosolic SR Ca^2+^ that might result from digitalis toxicity and catecholaminergic polymorphic ventricular tachycardia (Kass & Tsien, 1982; Marks *et al.* 2002; Bers, 2008; Sabir *et al.* 2008*a*).

The subsequent experiments in intact hearts accordingly proceeded to explore the effects of pretreatments using three different pharmacological agents, the diydropyridine nifedipine, the benzothiazipine diltiazem and the indole tetramic acid CPA on arrhythmogenic tendency induced by subsequent introduction of FPL (Saumarez & Grace, 2000; Saumarez *et al.* 2003). Nifedipine and diltiazem are L-type Ca^2+^ channel blockers, with diltiazem additionally inhibiting calcium leak through RyR2s. Previous experiments using PES protocols showed that both these agents block triggered VT induced by either caffeine or isoproterenol (Balasubramaniam *et al.* 2003, 2004). In contrast, CPA is a potent and specific inhibitor of SERCA-mediated Ca^2+^ uptake (Goeger *et al.* 1988; Mason *et al.* 1989) without affecting Ca^2+^ sensitivity of the contractile apparatus (Takahashi *et al.* 1995), Ca^2+^ currents (Bonnet *et al.* 1994; Badaoui *et al.* 1995) and the NCX (Yard *et al.* 1994). It has been used to investigate the role of SERCA in regulating the relationship between contraction, intracellular Ca^2+^ transients and force–frequency relationships (Goeger *et al.* 1988; Mason *et al.* 1989; Baudet *et al.* 1993; Schwinger *et al.* 1997).

These experiments demonstrated that these effects of FPL could be attributed to its enhancing extracellular Ca^2+^ entry; FPL-induced arrhythmogenesis was abolished in hearts subjected to pretreatment with either nifedipine or diltiazem. The effects were also dependent upon the degree of filling of the SR Ca^2+^ store, and accordingly abolished by CPA. Furthermore, none of these agents, whether in the presence or absence of FPL, altered APD values, in contrast with earlier reports of the actions of such agents in LQT3 (Killeen *et al.* 2007*a*; Thomas *et al.* 2007*a*).

In contrast, these arrhythmic effects correlated with alterations in Ca^2+^ homeostasis at the single-cell level. These experiments investigated the effects of FPL, as well as nifedipine, diltiazem and CPA, upon regularly stimulated fluo−3 loaded myocytes subjected to similar pharmacological manoeuvres to those used in the whole hearts. Thus, FPL alone resulted in an immediate increase in peak evoked *F*/*F*_0_ as well as an onset of diastolic Ca^2+^ transients in isolated myocytes subjected to regular stimulation. This change was dependent upon a Ca^2+^ entry enhanced by FPL. Thus, both nifedipine and diltiazem reduced such transients, whose amplitude was then not restored by a subsequent administration of FPL; in such conditions, diastolic Ca^2+^ transients were absent. Similarly, myocytes pretreated with CPA had reduced peak *F*/*F*_0_ transients. These were not restored by a subsequent addition of FPL. Nevertheless, addition of FPL resulted in an appearance of diastolic transients whose amplitude was substantially lower than diastolic transients observed with FPL alone. This is as expected for an action of FPL in activating cardiac RyR2 either by Ca^2+^ entry or through a direct effect reported earlier (Wasserstrom *et al.* 2002; McDonough *et al.* 2005).

The present findings are thus compatible with a major role for extracellular Ca^2+^ entry in this arrhythmogenecity that is attributable to a gradually developing intracellular calcium overload.
